# A 1 week IGF‐1 infusion decreases arterial insulin concentrations but increases pancreatic insulin content and islet vascularity in fetal sheep

**DOI:** 10.14814/phy2.13840

**Published:** 2018-09-02

**Authors:** Alicia White, Samantha Louey, Eileen I Chang, Brit H. Boehmer, David Goldstrohm, Sonnet S. Jonker, Paul J. Rozance

**Affiliations:** ^1^ Department of Pediatrics Perinatal Research Center University of Colorado Denver School of Medicine Aurora Colorado; ^2^ Center for Developmental Health Knight Cardiovascular Institute Oregon Health & Science University Portland Oregon

**Keywords:** Beta‐cell, fetus, insulin, insulin‐like growth factor, islet

## Abstract

Fetal insulin is critical for regulation of growth. Insulin concentrations are partly determined by the amount of *β*‐cells present and their insulin content. Insulin‐like growth factor‐1 (IGF‐1) is a fetal anabolic growth factor which also impacts *β*‐cell mass in models of *β*‐cell injury and diabetes. The extent to which circulating concentrations of IGF‐1 impact fetal *β*‐cell mass and pancreatic insulin content is unknown. We hypothesized that an infusion of an IGF‐1 analog for 1 week into the late gestation fetal sheep circulation would increase *β*‐cell mass, pancreatic islet size, and pancreatic insulin content. After the 1‐week infusion, pancreatic insulin concentrations were 80% higher than control fetuses (*P* < 0.05), but there were no differences in *β*‐cell area, *β*‐cell mass, or pancreatic vascularity. However, pancreatic islet vascularity was 15% higher in IGF‐1 fetuses and pancreatic *VEGFA*,*HGF*,*IGF1*, and *IGF2* mRNA expressions were 70–90% higher in IGF‐1 fetuses compared to control fetuses (*P* < 0.05). Plasma oxygen, glucose, and insulin concentrations were 25%, 22%, and 84% lower in IGF‐1 fetuses, respectively (*P* < 0.05). The previously described role for IGF‐1 as a *β*‐cell growth factor may be more relevant for local paracrine signaling in the pancreas compared to circulating endocrine signaling.

## Introduction

Insulin‐like growth factor 1 (IGF‐1) is an endocrine and paracrine growth factor with an important role in regulating *β*‐cell mass and production of insulin. In studies with adult animals and isolated pancreatic islets, IGF‐1 protects against loss of *β*‐cells following streptozotocin and interleukin‐1*β* exposure, although other studies demonstrate that IGF‐1 inhibits release of insulin from the pancreatic *β*‐cells (Leahy and Vandekerkhove 1990; Mabley et al. 1997; Porksen et al. 1997; Giannoukakis et al. 2000; Agudo et al. 2008; Robertson et al. 2008). While these studies show important effects of IGF‐1 on *β*‐cell mass and insulin production, the physiological endocrine regulation of fetal islet development and *β*‐cell mass are largely unexplored. Understanding this regulation is important because common complications of pregnancy result in abnormal *β*‐cell mass and islet development with implications for fetal insulin concentrations and the regulation of fetal growth (Green et al. 2010; Boehmer et al. 2017; Limesand and Rozance 2017). For example, fetuses from pregnancies complicated by placental insufficiency and severe intrauterine growth restriction (IUGR) are characterized by lower circulating IGF‐1 and lower *β*‐cell mass (Van Assche et al. 1977; Ostlund et al. 1997). Importantly, both fetal IGF‐1 and insulin are critical for appropriate regulation of fetal somatic growth (Fowden 1992, 2003), with cord blood concentrations of both insulin and IGF‐1 directly associated with birth weight including in large for gestational age newborns (Ostlund et al. 1997; Christou et al. 2001; Setia et al. 2006).

Pregnant sheep are used to model conditions of abnormal fetal growth and to test the basic physiological role of insulin and IGF‐1 in fetal growth and development (Fowden 2003; Barry and Anthony 2008). However, in a normally grown fetus, the impact of chronically increased circulating IGF‐1 at the end of gestation on fetal pancreatic islet size, structure, and *β*‐cell mass has not been tested. We hypothesized that an infusion of an IGF‐1 analog with very low affinity for binding proteins, IGF‐1 LR3, for 1 week into late gestation fetal sheep circulation would increase *β*‐cell mass, islet size, and pancreatic insulin content. Our results demonstrate that this experimental infusion results in higher fetal pancreatic insulin content. Contrary to our hypothesis, IGF‐1 LR3 does not result in higher *β*‐cell mass or larger pancreatic islets. However, although the fetal pancreatic islets were the same size following the IGF‐1 LR3 infusions compared to controls, they were more highly vascularized than control fetal islets. Furthermore, the IGF‐1 infused fetuses had mRNA expression of pancreatic *VEGFA*,* HGF*,* IGF1,* and *IGF2* that was 70–90% higher than expression in control pancreases.

## Materials and Methods

### Fetal sheep and experimental protocol

Studies were conducted in time bred mixed Western breed sheep at Oregon Health & Science University in Portland, Oregon with approval of the Institutional Animal Care and Use Committee. This center is accredited by the Association for Assessment and Accreditation of Laboratory Animal Care International (AAALAC). Sheep were obtained from a local vendor (Agna LLC). Ewes were maintained on a 12 h light/dark cycle in a social housing environment with soft bedding, and allowed ad lib access to food (flake alfalfa, pelleted alfalfa, and “corn, oats, barley with molasses” [C.O.B.]) and water. They were acclimatized to the facility for 3–7 days prior to surgery.

At 120 ± 0 days gestational age (term ~147 days), fetal catheters were surgically placed similar to previously described (Jonker et al. 2016). In brief, to control secretions ewes were given intramuscular atropine (7.5 mg), anesthetized with intravenous ketamine (400 mg) and diazepam (10 mg), and ventilated with oxygen:nitrous oxide (2:0.7) and isoflurane (1.5–2.0%). Fetuses were exteriorized through a uterine incision for catheter placement prior to closing in layers. Ciprofloxacin (2 mg) and Penicillin G (1 million units) were injected into the fetal amniotic sac at the conclusion of surgery. Ewes received subcutaneous buprenorphine (0.3 mg) immediately after surgery and twice daily for 2 days thereafter during surgical recovery. Following surgery they were monitored for full recovery, including resumption of normal posture, food and water consumption, urination and defecation, behavior with conspecifics, and responsiveness to human interaction. When recovered, animals were acclimatized to metabolic cages, in which they had ad libitum food and water and could stand or lie.

Beginning 6 ± 0 days after surgery, fetuses were randomized to receive a chronic constant infusion of IGF‐1 LR3 (6.6 *μ*g·kg^−1^·h^−1^; *n* = 8) or saline (volume matched, ~30 mL·day^−1^; *n* = 10) as a control infusion for 7 days. This compound was selected to test the effects of IGF‐1 independent of its effect when associated with binding proteins, as it associates with binding proteins at ~1000‐fold lower affinity. The dose was selected based on its action in the fetus (Sundgren et al. 2003). In the case of a twin pregnancy only one fetus was randomized and studied. The larger twin based on estimated body size at the time of surgery was selected for instrumentation and study. After the fetal blood was sampled on day 7, and prior to necropsy, all fetuses were subjected to a ~1 h hypoxic hemodynamic challenge, during which time maternal oxygen saturation was reduced to 50% by tracheal nitrogen infusion, followed by 15 m recovery prior to necropsy, as part of an experiment not reported in this manuscript.

### Biochemical analysis

Daily fetal arterial plasma glucose and lactate concentrations, pH, partial pressure of oxygen (PaO_2_), partial pressure of carbon dioxide (PaCO_2_), and oxygen concentration were measured with the blood gas analyzer ABL 825 (Radiometer America Inc., Westlake, OH, USA), and the hematocrit was measured from microcentrifugation of capillary tubes. Fetal hormones were measured on day 0 and day 7. Arterial plasma insulin and cortisol were measured by enzyme‐linked immunosorbent assay (ELISA) (insulin: intra‐ and interassay coefficients of variation = 4.3% and 4.7%, respectively; sensitivity = 0.14 ng/mL; cortisol: intra‐ and interassay coefficients of variation = 4.6% and 5.8%, respectively; sensitivity = 1.0 ng/mL; ALPCO, Salem, NH, USA). Arterial plasma norepinephrine was measured by high performance liquid chromatography (intra‐ and interassay coefficients of variation = 9.2% and 9.0%, respectively; sensitivity = 170 pg/mL; model 2475; Waters, Milford, MA) (Thorn et al. 2013).

### Pancreas collection

At the conclusion of the study, animals were euthanized with an intravenous overdose of a pentobarbital euthanasia solution. Fetal sex and weights were recorded. The pancreases were weighed and dissected into the splenic portion, processed for morphometric analysis, and the hepatic portion, which was snap frozen in liquid nitrogen and transferred to −80°C for mRNA and protein extraction.

### mRNA analysis

mRNA was extracted from pulverized −80°C pancreas (100 mg) and reverse transcribed into complimentary DNA (cDNA) as previously described (IGF‐1, *n* = 6; control, *n* = 5) (Benjamin et al. 2017). Quantitative PCR assays were performed on a 1:10 dilution (with sterile water) of the reverse transcription cDNA for *INS* (insulin), *GCG* (glucagon), *SST* (somatostatin), *PPY* (pancreatic polypeptide), *PDX1* (pancreatic and duodenal homeobox‐1), *GCK* (glucokinase), *SLC2A2* (glucose transporter‐2 [GLUT‐2]), *IGF1* (insulin‐like growth factor‐1 [IGF‐1]), *IGF2* (IGF‐2), *VEGFA* (vascular endothelial growth factor), *HGF* (hepatocyte growth factor), and *RPS15* (ribosomal protein S15) were performed and validated as previously described (Rozance et al. 2007; Chen et al. 2012; Gadhia et al. 2013; Lavezzi et al. 2013; Brown et al. 2016). The total volume of each reaction was 10 *μ*L: 4 *μ*L cDNA, 0.5 *μ*L for each primer (0.5 mol/L), and 5 *μ*L Faststart Universal SYBR Green MM (Roche, San Francisco, CA, USA). The temperature cycles were 5 min at 95°C to activate the enzyme; then 40 cycles of 30 sec at 60°C, 30 sec at 72°C, and 10 sec at 96°C. To verify the correct amplification of the reaction, we examined the melt curve analysis to ensure a single peak. The cDNA samples were analyzed in triplicate and the standard curve method of relative quantification was utilized (Wong and Medrano 2005). Genes of interest were normalized to the reference gene *RPS15*, which was not different between groups. Results are presented relative to controls.

### Protein analysis

Protein was extracted from pulverized pancreas, separated by gel electrophoresis, and immunoblotting performed as described previously (IGF‐1, *n* = 7; control, *n* = 5) (Andrews et al. 2015). For immunoblotting, membranes were blocked in Tris‐buffered saline with 0.1% Tween 20 (v/v) and 5% nonfat dried milk (NFDM; w/v) prior to incubation with primary antibodies; rabbit anti‐glucokinase (1:1000; Abcam Inc., Cambridge, MA), rabbit anti‐GLUT2 (1:1000; Abcam Inc.), VEGFA (1:200; Santa Cruz Biotechnology, Dallas, TX), HGF (1:1000; R&D Systems, Minneapolis, MN), and mouse anti‐actin (1:10,000; MP Biomedicals, Salon, OH). Primary antibodies were diluted in Tris‐buffered saline with 0.1% Tween 20 (w/v) with 5% bovine serum albumin (BSA; v/v) except VEGFA which was incubated in 5% BSA (w/v) with 1% NFDM. Membranes were incubated with primary antibodies overnight and immunocomplexes detected with goat anti‐rabbit IRDye 800CW, donkey anti‐Goat IRDye 800CW, or goat anti‐Mouse IRDye 680RD (LI‐COR Inc., Lincoln, NE). Immunocomplexes were visualized and quantified using an Odyssey Fc imaging system (Image Studio; LI‐COR). Densitometry for proteins of interest were normalized to actin, which was not different between groups. Results are presented relative to controls.

For insulin content of the pancreas, three aliquots (between 12 and 25 mg each) of pulverized pancreas per fetus (IGF‐1, *n* = 7; control, *n* = 5) were subjected to an acid ethanol extraction with 1 mL of 1 mol/L HCl in 70% ethanol (v/v) at −20°C for 18 h (Rozance et al. 2007). The concentration of insulin was measured by ELISA (as described above). The three samples for each animal were averaged to obtain a single value.

### Histology of the fetal pancreas

Histological evaluation of the fetal pancreas (IGF‐1, *n* = 8; control, *n* = 7) was based on our previous protocols (Limesand et al. 2005; Rozance et al. 2007, 2015). Four 5 *μ*m thick sections were cut at 100 *μ*m intervals from each pancreas. Frozen sections were adapted to room temperature for 30 min prior to three, 5 min washes in deionized water. Sections were transferred to a 10 mmol/L citric acid buffer (pH 6.0) and maintained in a 90°C water bath for 30 min. Sections were cooled for 20 min, washed three times in phosphate buffered saline (PBS) for 10 min. Sections were blocked for 30 min in 1.5% normal donkey serum in PBS. Endocrine hormones were identified with guinea pig anti‐porcine insulin (1:250; Dako, Carpinteria, CA), mouse anti‐porcine glucagon (1:500; Sigma‐Aldrich, St. Louis, MO), rabbit anti‐human somatostatin (1:500; Dako), and rabbit anti‐human pancreatic polypeptide (1:500; Dako). Immunocomplexes were detected with the following affinity‐purified secondary antiserum (1:500): anti‐rabbit IgG conjugated to Cy2, anti‐mouse IgG conjugated to Texas Red, and anti‐guinea pig IgG conjugated to 7‐amino‐4‐methylcoumarin‐3‐acetic acid (AMCA; Jackson ImmunoResearch Laboratories, West Grove, PA). Fluorescent images were visualized on an Olympus IX83 microscope system (Olympus; Waltham, MA). Images were captured and morphometric analyses were performed using the cellSens software (Olympus). Insulin‐positive cells and glucagon‐positive cells were used to determine the *β*‐cell and *α*‐cell mass, respectively, by multiplying the pancreas weight by the percent total pancreas area positive for each hormone. The entire pancreatic section was used to determine *β*‐ and *α*‐cell mass.

Pancreatic and islet vessel density were measured as we have previously described (Rozance et al. 2015). Tissue sections were blocked with 0.5% NEN Block (Perkin‐Elmer, Waltham, MA) for 1 h, followed by addition of the anti‐insulin, glucagon, somatostatin, and pancreatic polypeptide primary antibodies noted above diluted in PBS with 1% BSA. FITC‐conjugated Griffonia simplicifolia (GS‐I) agglutinin (green, 15 *μ*g/mL; Vector Laboratories, Burlingame, CA) was added to the mixture of primary antibodies. Samples were incubated overnight at 4°C. Secondary antibodies were as described above except that Texas Red goat anti‐rabbit (1:500; Jackson ImmunoResearch Laboratories) replaced the CY2 goat anti‐rabbit IgG antibody. The entire pancreatic section was used to determine *β* pancreatic vessel density. Pancreatic islets were defined as clusters of endocrine cells with an area of >500 *μ*m^2^. An average of 119 ± 8 islets/animal were evaluated. Vessel density of all islets from one fetus were averaged to produce a mean value for use in summary and comparative statistics (Rozance et al. 2015).

### Statistical analysis

Statistical analysis was performed using SAS version 9.2 (SAS Institute). Results are expressed as mean ± Standard Error (SE). In a preliminary analysis we determined for all outcomes if fetal sex and singleton versus twin were significant. When significant, they were included in the final mixed‐model analysis of variance (ANOVA) analysis, which included terms (as appropriate) for treatment group (IGF‐1 or control), time (days of treatment), and interactions as main effects. A term was included to account for repeated measures made in the same animal. If the overall ANOVA was significant (*P* < 0.05), then Fishers protected least squares difference was used for post‐test comparisons. *P*‐values ≤ 0.05 were accepted as significant. If data were found not to be normally distributed or to have unequal variance between IGF‐1 and control groups, data were either analyzed by the Mann–Whitney test or log transformed for ANOVA.

## Results

At the end of the study, gestational ages were similar in both groups (134 ± 0 days, control vs. 133 ± 0 days, IGF‐1). The proportion of male fetuses (50%, control vs. 62.5%, IGF‐1) and twin pregnancies (20%, control vs. 50%, IGF‐1) also were statistically similar in both groups. IGF‐1 fetuses were heavier than the control fetuses (3.86 ± 0.25 kg, control vs. 4.42 ± 0.14 kg, IGF‐1; *P *≤ 0.05, Mann–Whitney test).

### Fetal biochemistry

**Figure 1:**
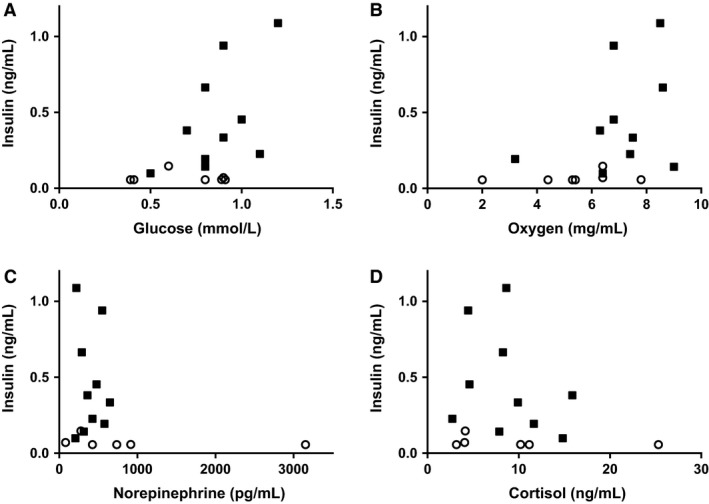
There was no correlation between fetal arterial plasma insulin concentrations in the IGF‐1 group with fetal arterial plasma glucose (A), oxygen (B), norepinephrine (C), or cortisol (D) concentrations. Individual control (■) and IGF‐1 (○) fetal data are plotted.

Fetal arterial plasma insulin and glucose concentrations decreased during the 7‐day infusion period in the IGF‐1 fetuses but not in the controls (*P* < 0.0005, Table 1). The decrease for insulin was greater than the decrease for glucose and so the insulin:glucose ratio was lower in the IGF‐1 fetuses compared to controls at the end of the experiment (*P* < 0.005, Table 1). Fetal arterial plasma lactate and blood arterial pH, PaCO_2_, and hemoglobin were not different between groups (Table 1). Although one animal in the IGF‐1 group had a fetal plasma arterial norepinephrine concentration over 3000 pg/mL, there was no statistical difference in norepinephrine concentrations between control and IGF‐1 groups whether or not this animal was included in the analysis (Table 1). Fetal arterial blood PaO_2_ and O_2_ content decreased in the IGF‐1 fetuses during the 7‐day infusion but not in the controls (*P* < 0.001, Table 1). Fetal arterial cortisol increased similarly in both groups (Table 1). Because low glucose and oxygen concentrations and high norepinephrine concentrations can inhibit insulin secretion (Dionne et al. 1993; Limesand and Hay 2003; Chen et al. 2014; Macko et al. 2016), we graphed fetal insulin concentrations with each of these. In no case could a relationship between glucose, oxygen or norepinephrine, and insulin concentrations in the IGF‐1 group be detected (Fig. 1).

**Table 1:** Fetal arterial plasma hormones, glucose, lactate, protein and blood pH, gases, and hematocrit before and after 7 days of IGF‐1 or control infusions

Parameter	Day 0		Day 7		*P*‐value			
	Control	IGF‐1	Control	IGF‐1		Trt	Day	Trt x Day
Insulin (ng/ml)†	0.32 ± 0.10	0.29 ± 0.08	0.45 ± 0.11	0.07 ± 0.01	*	**<0.005**	**<0.05**	**<0.0005**
Glucose (mmol/L)	0.9 ± 0.1	1.0 ± 0.1	0.9 ± 0.1	0.7 ± 0.1	*	**<0.05**	**<0.0001**	**<0.0001**
Insulin:Glucose (ratio)†	0.32 ± 0.08	0.29 ± 0.07	0.50 ± 0.10	0.11 ± 0.03	*	**<0.005**	0.80	**<0.005**
Lactate (mmol/L)	1.54 ± 0.10	1.48 ± 0.0.9	1.54 ± 0.10	2.69 ± 0.98		0.07	0.17	0.18
pH	7.35 ± 0.01	7.34 ± 0.01	7.32 ± 0.01	7.32 ± 0.01		0.74	**<0.05**	0.16
pCO_2_ (mmHg)	51.4 ± 1.1	51.2 ± 0.8	51.6 ± 1.0	54.1 ± 0.7		0.28	0.18	0.19
pO_2_ (mmHg)	19.9 ± 0.9	20.4 ± 0.6	19.9 ± 0.5	17.0 ± 0.9	*	**<0.005**	**<0.05**	**<0.01**
Hemoglobin (gm/dL)	9.8 ± 0.5	10.6 ± 0.5	10.0 ± 0.7	9.6 ± 0.7		0.79	0.39	0.14
O_2_ content (mg/dL)	7.2 ± 0.3	7.9 ± 0.5	7.1 ± 0.5	5.3 ± 0.6	*	**<0.05**	**<0.005**	**<0.005**
Cortisol (ng/ml)†	5.6 ± 0.5	4.4 ± 0.7	8.9 ± 1.4	10.1 ± 2.9		0.98	**<0.005**	0.37
Norepinephrine (pg/ml)^#^, †	434 ± 23	451 ± 121	406 ± 49	887 ± 392		0.72	0.52	0.23

All data obtained throughout days zero through seven were analyzed by mixed model ANOVA with repeated measures and presented as means ± SEM from control (*n* = 10) and IGF‐1 (*n* = 7^†^ or 8) animals. Partial pressure CO_2_ (pCO_2_), partial pressure O_2_ (pO_2_).

^#^Data were log transformed for analysis.

*Indicates *P* < 0.005 by Fishers protected least squares difference test between IGF‐1 and control fetuses on day 7.

### Characteristics of the fetal pancreas

**Figure 2:**
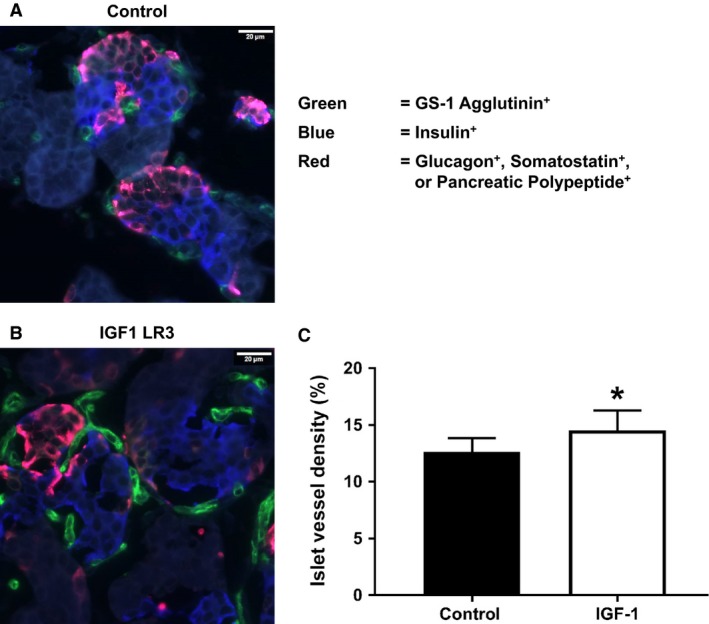
Pancreatic islet vessel density was measured by immune‐ and GITC‐conjugated GS‐1 staining and was higher in IGF‐1 fetuses (B) compared to Control fetuses (A). Costatining was performed for insulin (blue) as well as glucagon, somatostatin, and pancreatic polypeptide (red). (C) Data are mean ± SE for Control (*n* = 7) and IGF‐1 (*n* = 8) fetuses. *Indicates *P* < 0.05 by Students *t* test.

Fetal pancreatic weights were not statistically different between the groups (Table 2). There were no differences between groups for the proportion of the pancreatic sections staining insulin‐positive cells or glucagon‐positive cells or for pancreatic *β*‐ or *α*‐cell mass (Table 2). There also were no differences for islet size or pancreatic vessel density. However, islet vessel density was 15% higher in the IGF‐1 pancreases (*P* < 0.05, Fig. 2). The pancreatic insulin protein content was 80% higher in IGF‐1 fetuses compared to control (*P* < 0.05), though there was no difference in pancreatic insulin mRNA expression, or the expression of the other major pancreatic islet endocrine hormones, glucagon, pancreatic polypeptide or somatostatin (Table 3). In order to explore the reason for lower arterial insulin concentrations in the IGF‐1 group, we measured pancreatic GLUT2 and glucokinase, and although pancreatic GLUT2 mRNA expression was 145% higher in the IGF‐1 group, we did not find differences in GLUT2 protein or glucokinase protein and mRNA expression (Table 3). We also determined the impact of the IGF‐1 treatment on pancreatic paracrine growth factors and found significantly higher mRNA concentrations of VEGFA, HGF, IGF‐1, and IGF‐2 (*P* < 0.05, Table 3). We did not find a corresponding increase in the pancreatic protein concentrations of VEGFA or HGF (Table 3).

**Table 2:** Histological characteristics of the fetal pancreas

	Control	IGF‐1
Pancreas weight (gm)	3.50 ± 0.24	3.62 ± 0.27
Islet size (*μ*m^2^)	2957 ± 114	3080 ± 256
Insulin‐positive area (%)	4.66 ± 0.65	5.20 ± 0.50
Glucagon‐positive area (%)	1.81 ± 0.21	1.51 ± 0.45
*β*‐cell mass (mg)	178 ± 31	187 ± 18
*α*‐cell mass (mg)	68 ± 8	51 ± 16
Pancreatic vessel density (%)	4.4 ± 0.2	4.3 ± 0.2

Values are expressed as means ± SE. *n* = 7 for control and *n* = 8 for IGF‐1, except for pancreas weight (*n* = 10 for control and *n* = 8 for IGF‐1).

**Table 3:** Molecular characteristics of the fetal pancreas

	Control	IGF‐1
Pancreatic insulin (*μ*g/gm)	114 ± 35	205 ± 18*
Pancreatic protein (fold change)		
Glucokinase	1.00 ± 0.37	0.86 ± 0.16
GLUT2	1.00 ± 0.40	0.57 ± 0.18
VEGFA	1.00 ± 0.21	1.36 ± 0.16
HGF	1.00 ± 0.23	0.90 ± 0.23
Pancreatic mRNA (fold change)		
Insulin	1.00 ± 0.18	1.10 ± 0.17
Glucagon	1.00 ± 0.25	0.66 ± 0.12
Pancreatic polypeptide	1.00 ± 0.25	1.53 ± 0.21
Somatostatin	1.00 ± 0.18	1.37 ± 0.17
PDX1	1.00 ± 0.08	1.14 ± 0.07
Glucokinase	1.00 ± 0.14	0.68 ± 0.24
GLUT2	1.00 ± 0.15	2.45 ± 0.35**
IGF‐1	1.00 ± 0.34	1.89 ± 0.19*
IGF2	1.00 ± 0.25	1.90 ± 0.19*
VEGFA	1.00 ± 0.07	1.80 ± 0.16**
HGF	1.00 ± 0.27	1.73 ± 0.21*

Values are expressed as means ± SE. *n* = 5 for control and *n* = 6 (mRNA) or 7 (protein) for IGF‐1. *, ** indicates *P* < 0.05, <0.005 by Student's *t* test.

## Discussion

In the present study, we show that fetal infusion of IGF‐1 LR3 for 1 week near the end of gestation results in higher fetal pancreatic insulin content but lower fetal arterial insulin concentrations. There was no change in pancreatic islet size or *β*‐cell mass. We also demonstrate for the first‐time that experimental infusion of an IGF‐1 analog results in higher vascularization of the pancreatic islets compared to a control group. This is consistent with higher pancreatic mRNA expression of VEGFA and HGF. VEGFA and HGF are two paracrine growth factors highly involved in establishing the vascularity of the pancreatic islet and mediating the stimulatory effect of the endothelial cells on *β*‐cell functions, like insulin production. We also found that following IGF‐1 LR3 infusions fetal body weights, but not pancreatic weights, were heavier. Thus, the previously described role for IGF‐1 as a *β*‐cell growth factor may be more relevant for local paracrine action in the pancreas as opposed to circulating endocrine actions. This is in contrast to the growth promoting effect of circulating IGF‐1 on other fetal tissues (Eremia et al. 2007; Fowden and Forhead 2009).

Fetal arterial plasma insulin and glucose concentrations decreased during the 7‐day infusion period in the IGF‐1 fetuses but not in the controls. While experimentally lowering fetal glucose concentrations results in lower circulating fetal insulin concentrations (Limesand and Hay 2003), in the IGF‐1 fetuses it is unlikely that lower insulin concentrations were caused by lower glucose concentrations for two reasons. First, the decrease for insulin was much greater than the decrease for glucose resulting in lower insulin to glucose ratios in the IGF‐1 fetuses compared to controls. Furthermore, inspection of panel A in Figure 1 shows that while the expected positive association between glucose and insulin concentrations exists in the control fetuses, in the IGF‐1 group there is no association. In fact, all the insulin concentrations in the IGF‐1 group are very low irrespective of the fetal glucose concentration. Put another way, even in the IGF‐1 fetuses with normal glucose concentrations of approximately 0.9 mmol/L, the insulin concentrations are all extremely low.

Fetal arterial blood PaO_2_ and O_2_ content also decreased in the IGF‐1 fetuses during the 7‐day infusion but not in the controls. Fetal hypoxemia can directly inhibit fetal insulin secretion as well as indirectly via increased fetal production of norepinephrine (Yates et al. 2012; Macko et al. 2016). In the current study, only a single IGF‐1 animal had elevated norepinephrine concentrations. Thus, the role of elevated norepinephrine concentrations in suppressing insulin concentrations in the entire IGF‐1 cohort of fetuses is likely to be minimal. Furthermore, similar to the situation with the relationship between fetal glucose and insulin concentrations in the current study, it is unlikely that lower insulin concentrations were caused by lower oxygen concentrations. Panels B and C in Figure 1 show that even in fetuses with normal arterial oxygen and norepinephrine concentrations, fetal insulin concentrations were low. This is consistent with our data from a chronic fetal anemic hypoxemia model in which we lowered fetal arterial oxygen concentrations even further than in the current study and found no change in circulating fetal insulin concentrations (Benjamin et al. 2017). Further supporting a direct effect of infused IGF‐1 causing lower insulin concentrations in our study is the observation that in adult humans and isolated adult rat pancreases, IGF‐1 is a potent inhibitor of glucose stimulated insulin secretion independent of low glucose concentrations (Leahy and Vandekerkhove 1990; Porksen et al. 1997). Thus, while there may be some impact of lower fetal plasma glucose and oxygen concentrations on insulin concentrations, it is likely that increased IGF‐1 endocrine signaling had a greater effect on lowering insulin secretion.

Unlike the impact on insulin concentrations, we did not find any impact of the IGF‐1 infusion on the proportion of the fetal pancreatic sections staining insulin‐positive or the *β*‐cell mass. Furthermore, we found that there were no differences between groups for islet size. However, the vascularization of the IGF‐1 islets was higher than control islets. The interactions between vascular endothelial cells and *β*‐cells are mediated by several cell‐cell signaling pathways between (Peiris et al. 2014). These pathways include the paracrine growth factors VEGFA and HGF, which we have shown are particularly important for fetal sheep pancreatic islet development and function in cases of impaired fetal growth (Rozance et al. 2015; Rozance and Hay 2016).

Both VEGFA and HGF showed significantly higher mRNA expression in the IGF‐1 pancreases. *β*‐cells express VEGFA, which interacts directly with endothelial cells to increase vascularization of the islet (Rozance and Hay 2016). It is possible that circulating IGF‐1 increased *β*‐cell expression of VEGFA, which in turn stimulated an increase in islet vascularity. Previous work in carcinoma cells has demonstrated the capacity for IGF‐1 to stimulate VEGFA mRNA expression (Warren et al. 1996; Fukuda et al. 2002), as we have proposed for fetal pancreatic islets (Rozance and Hay 2016). We acknowledge that the changes in pancreatic mRNA expression may have been related to the short in vivo hemodynamic challenge these animals were subjected to prior to collection of the fetal pancreas. However, the challenge was the same in both groups of fetuses and therefore, our results still show the tendency for higher VEGF and HGF mRNA expression in the IGF‐1 fetuses, whether due directly to the IGF‐1 LR3 or to the brief hemodynamic challenge. Experiments with isolated fetal sheep pancreatic islets are now underway in our laboratory to confirm the relationship between IGF‐1 and VEGFA in vitro. Support for the capacity for IGF‐1 to increase islet vascularization via a *β*‐cell VEGFA pathway also comes from the observation that it was only islet vascularity that increased in the IGF‐1 fetuses; total pancreatic vascularity was not different between groups.

Islet endothelial cells signal to *β*‐cells to increase various *β*‐cell functions, including the synthesis and storage of insulin. Thus, the higher pancreatic insulin concentrations of the IGF‐1 fetuses may be an indirect effect mediated through increased islet vascularity. One of the primary pathways by which islet endothelial cells stimulate *β*‐cells is through secretion of the paracrine growth factor, HGF (Rozance and Hay 2016). We have previously demonstrated in isolated fetal sheep pancreatic islets the stimulatory effect that HGF has on *β*‐cell production and storage of insulin (Rozance et al. 2015). Alternatively, IGF‐1 might directly stimulate *β*‐cell insulin production and storage in fetal sheep islets, a possibility currently being tested in our laboratory using isolated fetal sheep pancreatic islets. It is interesting that despite an increase in pancreatic VEGFA, HGF, IGF1, and IGF2 mRNA expression, islet size was not different between IGF‐1 infused and control fetuses. Our work in a model of placental insufficiency and fetal growth restriction supports the assertion that islet size and vascularity are linked, and that changes in islet size may lag behind changes in islet vascularity (Rozance et al. 2015; Brown et al. 2016). Thus, it is possible that a longer exposure to elevated circulating IGF‐1 would result in larger pancreatic islets.

Overall growth in the late gestation sheep fetus is strongly reliant on the interplay of several nutrients and hormones (Fowden 1992, 2003; Bauer et al. 1998; Fowden and Forhead 2009). IGF‐1 and insulin are two of the most important anabolic fetal growth factors, and have overlapping mechanisms of action (Fowden 1992, 2003). Concentrations of both hormones in cord blood are directly correlated to size at birth, and genetic absence of these hormones results in IUGR in humans (Lemons et al. 1979; Woods et al. 1996; Ostlund et al. 1997; Christou et al. 2001; Setia et al. 2006). However, these two hormones do not act independent of each other. In addition to overlap in receptor binding (Fowden 2003; Kulkarni 2005), we have previously demonstrated that chronic infusion of insulin into the fetal circulation increases arterial IGF‐1 concentrations by 30% (Andrews et al. 2015). Combined with our current study demonstrating a significant reduction in circulating insulin concentrations following chronic infusion of the IGF‐1 agonist and other studies (Fowden and Forhead 2009), it is clear these two anabolic hormones have a complex relationship for regulating each other and for regulating fetal growth.

Testing the role of IGF‐1 on the developing fetal pancreatic islet is important to gain a better understanding of the regulation of fetal *β*‐cell mass and insulin secretion in fetuses exposed to certain pregnancy complications which result in abnormal fetal growth (accelerated or impaired), which is directly related to fetal IGF‐1 concentrations (Ostlund et al. 1997; Christou et al. 2001). Given the fact that many of these complications also are associated with abnormal pancreatic islet development and function, determining the exact mechanisms responsible for abnormal pancreatic islet development in these conditions requires a more basic understanding of the role of IGF‐1 in this process, as provided by our current set of data. Furthermore, our physiological studies with IGF‐1 fetal infusions may also have important implications for the care of prematurely born infants in which chronic infusions of a different IGF‐1 complex has been considered as a preventative therapy for retinopathy of prematurity, bronchopulmonary dysplasia, and to improve brain and body growth in preterm infants (Hellström et al. 2016).

## Conflict of Interest

The authors have nothing to disclose and no competing interests.
